# qPCR-Based Reference Gene Validation in *Canarium album*: Stability Across Varieties and Developmental Stages

**DOI:** 10.3390/cimb47110903

**Published:** 2025-10-30

**Authors:** Qingqing Zhao, Lai Jiang, Wenbao Luo, Wei Wang, Chaogui Shen, Qinghua Ye, Qingxi Chen, Qian Xie

**Affiliations:** 1 College of Horticulture, Fujian Agriculture and Forestry University, Fuzhou 350002, China; qingqingzhao1002@163.com (Q.Z.);; 2 Fruit Research Institute, Fujian Academy of Agricultural Sciences, Fuzhou 350013, China; 3 College of Horticulture and Landscape Architecture, Fujian Vocational College of Agriculture, Fuzhou 350303, China

**Keywords:** *Canarium album*, fruit, reference gene, qRT-PCR, stability evaluation

## Abstract

To obtain stable Chinese olive reference genes, eight genes (*RPN2B*, *PIP1.4*, *NIFS1*, *RPS16*, *At5g12110*, *HSC-2*, *ABCG44*, *LOS1*) exhibiting stable expression were identified as candidate reference genes from the transcriptome. The expression stability of these genes was evaluated across 33 Chinese olive fruit samples from different varieties and seven developmental stages. The most stable reference genes were determined through comparisons using ΔCq, geNorm, NormFinder, BestKeeper, and RefFinder. Analysis revealed that *RPN2B* and *NIFS1* were consistently ranked among the most stable genes across the different algorithms and exhibited stable expression. Therefore, they are recommended as suitable reference genes for gene expression studies in Chinese olive fruits across different varieties and developmental stages. The four different methods of reference gene stability analysis were used to identify the most stable reference genes in different varieties and developmental stages of Chinese olive fruits, which can be used as a reference for the selection of reference genes in the subsequent gene expression studies of Chinese olive fruits.

## 1. Introduction

Chinese olive (*Canarium album* L.) is a fruit tree belonging to the genus *Canarium* in the family Burseraceae, which is native to China and has been extensively cultivated in the provinces of Fujian, Guangdong, and Guangxi [1], with Fujian Province as the primary cultivation area [2]. Chinese olives are known for their unique flavor [3] and are rich in various nutritional and medicinal components [4]. Specifically, they contain high levels of vitamins [5], flavonoids [6], and other beneficial substances, seeing them classified as both medicinal and food fruits. They offer a range of pharmacological benefits and contribute significantly to human health. Gene expression analysis is a fundamental aspect of molecular biology research. Quantitative real-time PCR (qRT-PCR) is widely employed due to its high sensitivity and specificity [7]. This technique quantifies the expression of target genes and is among the most commonly used methods in contemporary molecular biology research, known for its precision, sensitivity, and speed [8]. Despite its many advantages, qRT-PCR requires appropriate normalization methods and reference genes with stable expression [9]. The stability of reference gene expression is crucial for qRT-PCR normalization and directly determines the reliability of experimental results [10].

Currently, the commonly used reference genes in plant qRT-PCR include *ACT*, *18S rRNA*, *GAPDH*, and *TUB*, among others [11,12]. Numerous experiments have demonstrated that these traditional reference genes exhibit relatively stable expression only under specific conditions. Their expression is not constant across various experimental factors, such as different species, developmental stages, and stress treatments [13]. Therefore, to obtain more reliable results in gene expression analysis, many researchers have screened for reference genes in a variety of plants, including *Pyrus bretschneideri* [9,14], *Cerasus pseudocerasus* [15], and *Cucurbita moschata* [16]. These studies have identified reference genes that can be stably expressed under diverse experimental conditions, developmental periods, and tissue types. As a perennial woody plant, the gene expression patterns in Chinese olives are significantly influenced by growth cycles and environmental factors. Traditional reference genes often do not exhibit stable expression across various species and developmental stages. Thus, screening for stably expressed reference genes in Chinese olive fruits is essential for enhancing the reliability of molecular biology studies in Chinese olives. The commonly used references demonstrate relatively stable expression under certain conditions; however, the instability of these reference genes across multiple varieties and developmental stages affects the accuracy of quantifying target gene expression in Chinese olive. Transcriptomic data analysis can identify genes that maintain stable expression across varieties and developmental stages; these genes may serve as potential reference genes. Software commonly used to evaluate reference genes includes geNorm [17], BestKeeper [18], and NormFinder [19]. Additionally, direct comparisons of Cq values and the use of integrated analysis tools like RefFinder [20] can be performed. Therefore, there is an urgent need for current research to systematically screen for broadly applicable reference genes in Chinese olive by combining these multiple bioinformatics evaluation methods.

The aim of this study was to identify reference genes that are stably expressed across various varieties and developmental stages of Chinese olive, utilizing transcriptome data screening and experimental validation. Initially, candidate reference genes were screened based on Chinese olive transcriptome data. qRT-PCR was employed to assess the expression levels of these candidate reference genes under diverse experimental conditions. The stability of the genes was evaluated through a combined analysis using multiple algorithms, including geNorm, NormFinder, BestKeeper, and RefFinder. The applicability of the optimal combinations of reference genes was confirmed by normalizing the expression of target genes. This study provides highly reliable reference genes for Chinese olive gene expression analysis, contributing significantly to the advancement of molecular biology research in this species.

## 2. Materials and Methods

### 2.1. Plant Materials

A total of thirty-three samples were collected for reference screening, along with eight samples for target gene verification, from various varieties (lines) of Chinese olive germplasm resources from the Chinese Olive Germplasm Resource Nursery located in Fuzhou City, Fujian Province, China (26°8′6″ N,119°15′58″ E) uniform management practices were implemented in an open-air environment. Chinese olive fruits from different varieties (lines) were harvested in mid-to late November 2023 after ripening (Table 1). The fruit development sampling period was divided into seven stages: 40 days, 55 days, 70 days, 95 days, 120 days, 155 days, and 190 days post-flowering. The varieties at different developmental stages were ‘A5’ and ‘A21’. Fruits were randomly collected from the east, west, south, and north aspects of each plant. Selected fruits, free from deformities and pest damage, were transported to the laboratory, rinsed with distilled water, peeled, pitted, thinly sliced, rapidly frozen in liquid nitrogen, and stored at −80 °C for subsequent experiments.

### 2.2. RNA Extraction and cDNA Synthesis

The samples were finely ground into a powder using liquid nitrogen, and 0.1 g of Chinese olive powder was utilized for RNA extraction, performed with the TIANGEN Polysaccharide Polyphenol Kit (TIANGEN, Beijing, China), according to the manufacturer’s instructions. The purity and integrity of the extracted RNA were assessed using 1% agarose gel electrophoresis (180 V, 16 min), and the RNA concentration was quantified using an ultra-micro spectrophotometer (Q5000, Quawell, San Jose, CA, USA). For cDNA synthesis, the ABclonal cDNA Synthesis Kit (ABclonal, Wuhan, China) was employed. Prior to the qRT-PCR, cDNA samples were purified and diluted 10-fold in nuclease-free ddH_2_O, and the resulting cDNAs were stored at −20 °C. Three biological replicates were performed, each consisting of three technical replicates.

### 2.3. Candidate Gene Selection and Primer Design

Candidate reference genes and target genes identified by transcriptome sequencing (https://ngdc.cncb.ac.cn/gsa/browse/CRA028315 (accesed on 15 September 2025)) of Chinese olive fruit. Genes with FPKM values below 40 failed to produce detectable bands in gel electrophoresis and were thus excluded from reference gene candidacy. Additionally, genes showing |log_2_FC| < 1 were considered not differentially expressed. Therefore, eight candidate reference genes fulfilling the criteria (FPKM ≥ 40 and |log_2_FC| < 1) were selected for further analysis. Given the established association of the starch and sucrose metabolism and phenylpropanoid biosynthesis pathways with key fruit characteristics and developmental stages, along with the observed differential expression of genes within these pathways, candidate target genes from these pathways were selected for validation, but amplification failed for some candidate genes. Ultimately, four target genes demonstrating stable amplification and the expected significant expression differences were selected for subsequent expression analysis. Primers for both the eight candidate reference genes (*RPN2B*, *PIP1.4*, *NIFS1*, *RPS16*, *At5g12110*, *HSC-2*, *ABCG44*, *LOS1*) and the four target genes (*TPS9*, *ISA3*, *PER64*, *CYP98A2*) were designed using Primer 3 Plus. These primers were subsequently synthesized by Fuzhou Sunya Biotechnology Co., Ltd. (Fuzhou, China). Details of the primers are provided in Table 2.

### 2.4. qRT-PCR and Amplification Efficiency Test

The qRT-PCR analyses were performed using a real-time fluorescence quantitative PCR instrument (qTOWER3, Analytik Jena AG, Jena, Germany) after loading samples into 96-well plates with a total reaction volume of 10 μL. This volume comprised 1 μL of diluted sample cDNA template, 5 μL of BrightCycle Universal SYBR Green qPCR Mix with UDG, 0.4 μL of primers, and 3.6 μL of ddH_2_O. The reaction program was set to 37 °C for 2 min, followed by 95 °C for 3 min, and then 40 cycles of 95 °C for 5 s and 60 °C for 34 s. Cq values and melting curves were obtained at the end of the process, with three biological replicates and three technical replicates per sample. Template cDNA was subjected to a 10-fold serial dilution to generate five concentrations, following the same reaction mixture composition and thermal cycling protocol as described above. Standard curves were prepared for each candidate reference gene, and the slope was derived from these standard curves. The amplification efficiency (E) of each primer was calculated using the formula E = 10^^(−1/slope)−1)^ × 100% [8].

### 2.5. Analysis of Expression Stability of Candidate Reference Genes

The expression stability of eight candidate reference genes was comprehensively analyzed using geNorm2003, NormFinder2004, BestKeeper2004 software, and the RefFinder online platform. Based on the relative quantitative data of Cq values for these reference genes, the average expression stability value (M) for each gene was calculated using geNorm software, employing the formula (1 + E)^−Cq^ (E represents the efficiency of amplification). Using the M value, geNorm ranks reference genes by stability: a lower M value indicates higher stability, while a higher M value indicates lower stability. Conversely, a higher M value suggests poorer stability. Additionally, geNorm also calculates pairwise variation (Vn/Vn + 1) to determine the optimal number of reference genes, where a smaller V value indicates better expression stability among gene pairs, making them suitable for use as reference genes. The default threshold for the V value is 0.15; if Vn/Vn + 1 < 0.15, then n is the most suitable number of reference genes. NormFinder calculates stability values from (1 + E)^−Cq^ data and ranks genes accordingly: the lowest stability value indicates the most stable reference gene. The criterion dictates that the gene with the smallest expression stability value is the most suitable reference gene, while the one with the highest stability value is deemed the least stable. BestKeeper evaluates stability using the raw Cq values, calculating the coefficient of variation (CV%) and standard deviation (SD). Lower CV% and SD values indicate higher stability of the reference gene. The specific operation refers to the method described by Pfaffl et al. [18]. Finally, the expression stability of the candidate reference genes was assessed by RefFinder (https://blooge.cn/RefFinder (accesed on 17 September 2025)), which integrates results from all four methods to generate a comprehensive stability ranking.

### 2.6. Validation of the Selected Candidate Reference Genes

The expression levels of four target genes (*TPS9*, *ISA3*, *PER64*, and *CYP98A2*) in Chinese olive fruits from different varieties and at different developmental stages were analyzed by qRT-PCR using the reference genes identified as optimal for normalization. The relative expression levels of the target genes were calculated using the 2^−ΔΔCq^ method [21].

## 3. Results

### 3.1. Primer Specificity Tests

**Figure 1:**
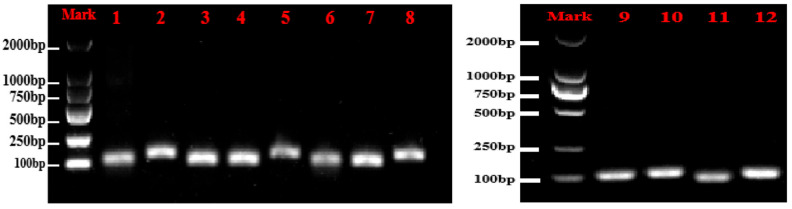
Amplification results of 12 genes with Chinese olives cDNA. 1~12, respectively, represent *RPN2B*, *PIP1.4*, *NIFS1*, *RPS16*, *At5g12110*, *HSC-2*, *ABCG44*, *LOS1*, *TPS9*, *ISA3*, *PER64*, and *CYP98A2*.

**Figure 2:**
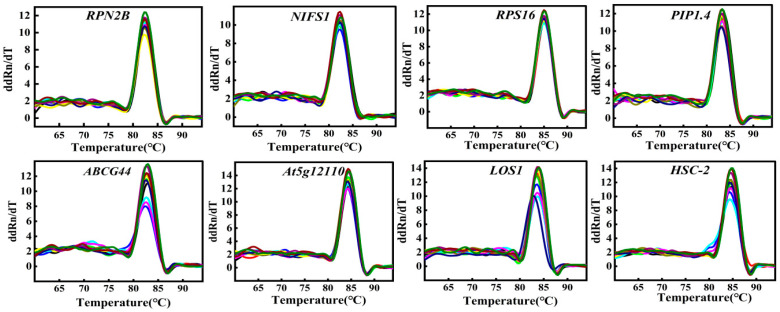
Melting curve of eight candidate reference genes in Chinese olive.

All primers had amplification efficiencies between 90 and 110% and R^2^ > 0.99 (Table 3), meeting the criteria for reliable analysis. Among the genes tested, *LOS1* exhibited the highest amplification efficiency, while *ABCG44* showed the lowest. These results indicate that the primer pairs performed effectively in experiments. Furthermore, the experimental data demonstrated a high degree of fit to the standard curves and exhibited high linearity. Figure 1 shows the amplified bands of 12 genes. Melting curve analysis revealed no non-specific products for individual single distinct peaks, which exhibited good repeatability. This demonstrates the specificity of the eight candidate reference genes (Figure 2).

### 3.2. Expression Analysis of Candidate Reference Genes

**Figure 3:**
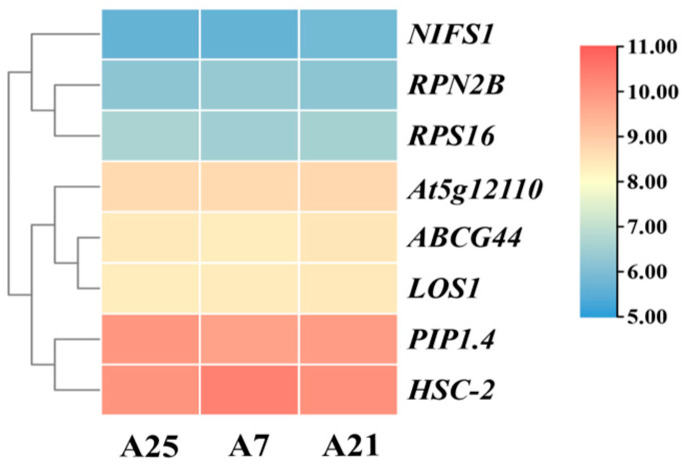
Trend plot of gene expression of 8 candidate reference genes in the transcriptome. A25, A7, and A21 are the 3 varieties at the time of transcriptome sequencing. Normalization was performed using log_2_ (FPKM + 1).

**Figure 4:**
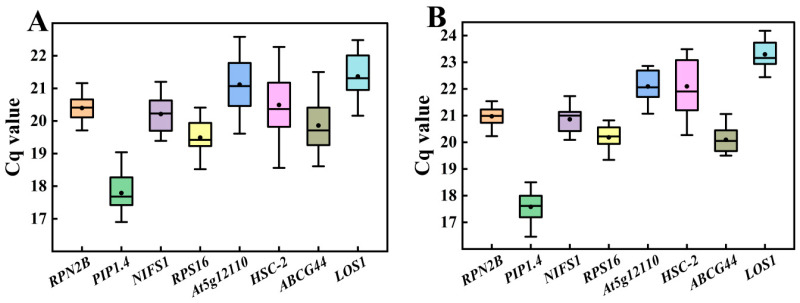
Cq values of eight candidate reference genes in Chinese olive fruits. (**A**) Different varieties; (**B**) Different developmental stages. The value for each sample is the mean of three technical replicates of three biological replicates, below. Boxes in the figure indicate 25–75% of the data, horizontal lines indicate the median, and black dots indicate the mean.

In the transcriptome database, a total of eight genes exhibiting stable expression were selected as candidate reference genes (Figure 3). qRT-PCR assays were performed on these eight genes in Chinese olive fruits at various developmental stages and among different varieties. The Cq values for the eight candidate reference genes were obtained, revealing that the expression levels of all genes fell within the range of 16–25, as illustrated in Figure 4. In Figure 4A, it is evident that the *RPN2B* and *RPS16* showed clustered Cq values *HSC-2* and *At5g12110* exhibited wider dispersion across different varieties. In contrast, when examining different developmental stages of the fruit (Figure 4B), *RPN2B* and *NIFS1* displayed a more concentrated distribution, whereas *HSC-2* and *At5g12110* remained the most dispersed.

### 3.3. geNorm Analysis

**Figure 5:**
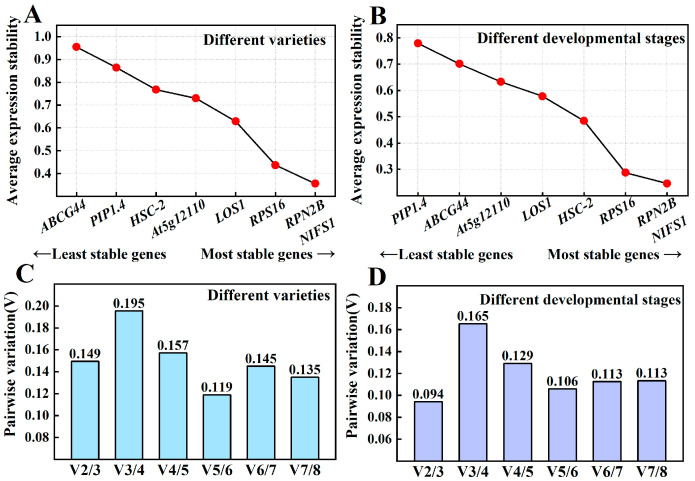
Average expression stabilization values in geNorm analysis of Chinese olive fruits. Average expression stability values of samples from different varieties (**A**) and developmental stages (**B**) in geNorm analysis; determination of the optimal number of reference genes in different varieties (**C**) and different developmental stages (**D**). Note: The next gene (*RPS16*) ranked third, with two genes tied for first place (*RPN2B*, *NIFS1*).

The two genes exhibiting the lowest M-value across various varieties were *RPN2B* and *NIFS1* (M = 0.356), followed closely by *RPS16* (M = 0.437). The M-values for these first three genes were relatively similar to each other but showed greater divergence from *LOS1* (M = 0.629), which ranked fourth. The gene with the highest M-value, indicating the most instability, was *ABCG44* (M = 0.955) (Figure 5A). Across different developmental stages, *RPN2B* and *NIFS1* maintained the lowest M-values (M = 0.246) and demonstrated the highest stability, followed by *RPS16* (M = 0.288). Conversely, the gene exhibiting the highest M-value and thus the greatest instability was *PIP1.4* (M = 0.780) (Figure 5B). In both contexts, *RPN2B* and *NIFS1* consistently ranked first, while *RPS16* ranked third. In the analysis of different varieties (Figure 5C), V2/3 was calculated as 0.149, which is less than 0.15, indicating stabilization upon the inclusion of the second reference gene, suggesting that the optimal number of reference genes is two. Similarly, in the context of different developmental stages (Figure 5D), V2/3 was determined to be 0.094, also less than 0.15, further confirming stabilization with the introduction of the second gene, confirming reinforcing that the optimum number of reference genes across different developmental stages is two. Overall, the optimal number of reference genes in both sample sets was determined to be two.

### 3.4. NormFinder Analysis

**Figure 6:**
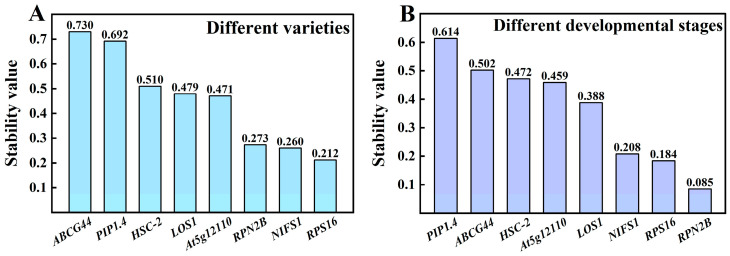
Expression stabilization values of NormFinder analysis in Chinese olive fruits. (**A**) Different varieties; (**B**) different developmental stages.

Among the various varieties (Figure 6A), the most stable reference gene was *RPS16*, with a stability value of 0.212, followed by *NIFS1* at 0.260 and *RPN2B* at 0.273. The stability values of these three genes were relatively close, while a significant difference was observed compared to the fourth-ranked gene, *At5g12110* (Stability value = 0.471). The least stable reference gene identified was *ABCG44*, with a stability value of 0.730. The stability ranking is as follows: *RPS16* > *NIFS1* > *RPN2B* > *At5g12110* > *LOS1* > *HSC-2* > *PIP1.4* > *ABCG44*. In different developmental stages (Figure 6 B), the most stable gene was *RPN2B*, with a stability value of 0.085, followed closely by *RPS16* at 0.184 and *NIFS1* at 0.208, showing minimal differences in their stability values. Conversely, the least stable gene identified was *PIP1.4*, with a stability value of 0.614, resulting in the following stability ranking: *RPN2B* > *RPS16* > *NIFS1* > *LOS1* > *At5g12110* > *HSC-2* > *ABCG44* > *PIP1.4*.

### 3.5. BestKeeper Analysis

As illustrated in Table 4, the order of stability across different varieties was *RPN2B* > *NIFS1* > *RPS16* > *PIP1.4* > *LOS1* > *At5g12110* > *HSC-2* > *ABCG44*, with *RPN2B* exhibiting the highest stability (1.810 ± 0.370), followed by *NIFS1* (2.080 ± 0.420) and *RPS16* (2.220 ± 0.430). The coefficient of variation (CV) values for all eight candidate reference genes were below 5, while the standard deviation (SD) values were all less than 1. Genes ranked in order of expression stability across various developmental stages were: *RPN2B* > *NIFS1* > *ABCG44* > *RPS16* > *LOS1* > *PIP1.4* > *At5g12110* > *HSC-2*. The gene with the lowest CV ± SD values was *RPN2B* (1.660 ± 0.350), whereas *HSC-2* had the highest (3.760 ± 0.830). Notably, the CV values for the eight candidate reference genes were all below 4, and the SD values were below 0.9. Thus, *RPN2B* and *NIFS1* were identified as the most stable reference genes across different varieties and developmental stages.

### 3.6. Overall Ranking Order and Selection of Best Reference Genes

**Figure 7:**
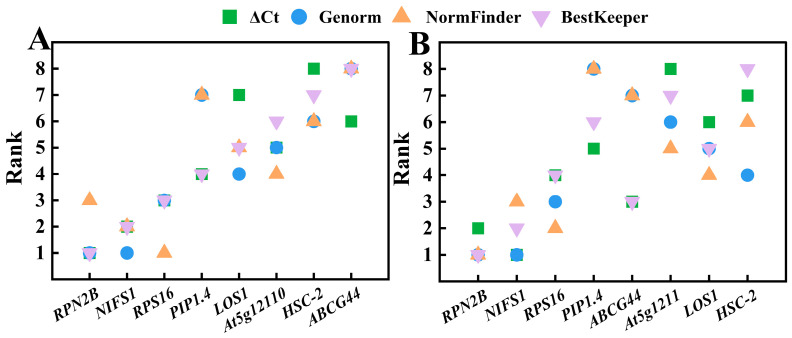
Ranking of stability values of eight candidate reference genes in Chinese olive analyzed by four methods. (**A**) Different varieties; (**B**) different developmental stages.

The stability of the eight reference genes was previously analyzed using four methods: direct comparison of ΔCq, geNorm, NormFinder, and BestKeeper. The optimal reference genes identified were inconsistent across these methods. To address this, the RefFinder online platform was utilized to obtain a comprehensive score for the eight candidate reference genes by analyzing their stability and ranking them (Table 5). A lower score indicates greater stability of the reference gene. Among the different varieties, RefFinder generated the following order of gene stability: *RPN2B* > *NIFS1* > *RPS16* > *PIP1.4* > *At5g12110* > *LOS1* > *HSC-2* > *ABCG44*. Notably, *RPN2B* ranked first with the lowest score (2.000), followed by *NIFS1* (2.646) and *RPS16* (2.659), indicating that these three genes exhibit the best expression stability across the different varieties. In contrast, *ABCG44* (8.000) and *HSC-2* (6.481) received higher composite scores, reflecting poorer stability. Among the four analytical methods (Figure 7A), *RPN2B* was ranked 3rd in NormFinder, while it ranked 1st in the others. *NIFS1* was ranked 1st in geNorm and 2nd in the other three methods, while *RPS16* was ranked 1st in NormFinder and 3rd in the other three methods. Consequently, *RPN2B* is the most stable gene across different varieties, followed by *NIFS1* and *RPS16*. The results obtained from the four analytical methods align with the stability ranking provided by RefFinder, confirming *RPN2B*, *NIFS1*, and *RPS16* as the top three most stable genes, in that order.

The order of gene stability generated by RefFinder across various developmental stages was *RPN2B* > *NIFS1* > *RPS16* > *LOS1* > *ABCG44* > *At5g12110* > *PIP1.4* > *HSC-2*, with *RPN2B* (1.000) identified as the most stable gene, followed closely by *NIFS1* (2.060) and *RPS16* (2.632). In contrast, *PIP1.4* (6.160) and *HSC-2* (7.113) exhibited the poorest stability. Among the four analysis methods (Figure 7B), *RPN2B* ranked second according to the ΔCq method, while the other three methods identified it as the most stable gene. The rankings for *NIFS1* were 1, 1, 2, and 3, whereas *RPS16* was ranked 2, 3, 4, and 4. Consistently, the rankings for *NIFS1* were superior to those of *RPS16*, which corroborates the results obtained from RefFinder. Therefore, the most stable reference genes across different developmental stages, in order, were: *RPN2B*, *NIFS1*, and *RPS16*.

Collectively, the most stable reference genes across various varieties and developmental stages were *RPN2B* and *NIFS1*, while the least stable reference gene was *ABCG44* for different varieties and *HSC-2* for different developmental stages.

### 3.7. Validation of Stability of Reference Genes

**Figure 8:**
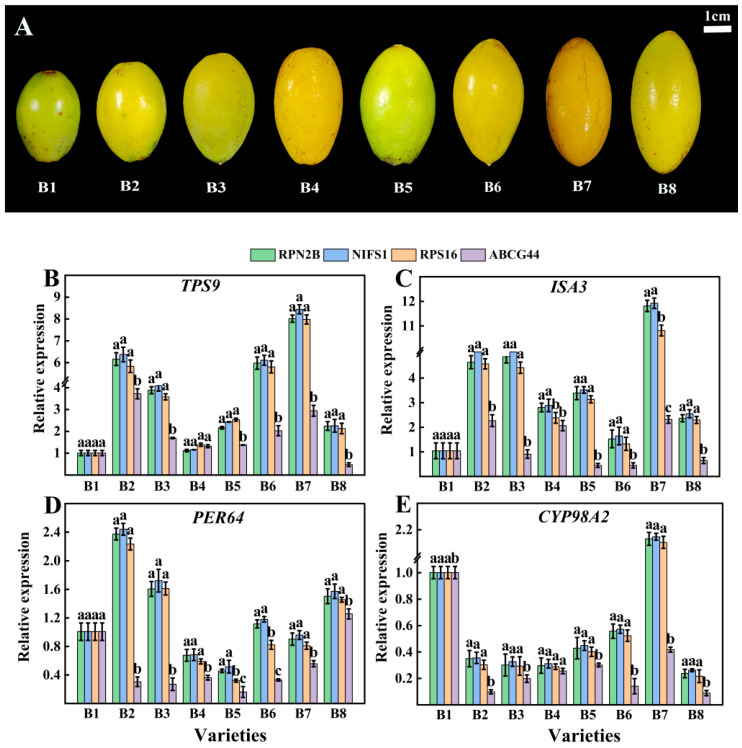
Validation of four target genes in different varieties of Chinese olive fruits with different reference gene stability. (**A**) Phenotypic characterization of eight validated varieties of Chinese olive; (**B**) validation in *TPS9*; (**C**) validation in *ISA3*; (**D**) validation in *PER64*; (**E**) validation in *CYP98A2*. Different letters within each column indicate significant differences between the means of the respective categories (*p* < 0.05).

**Figure 9:**
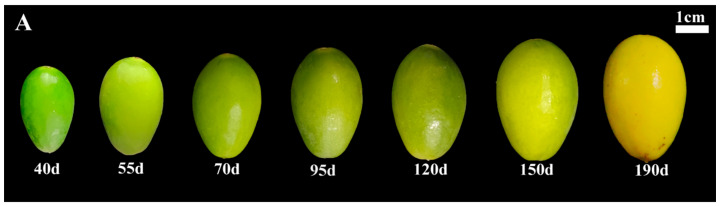

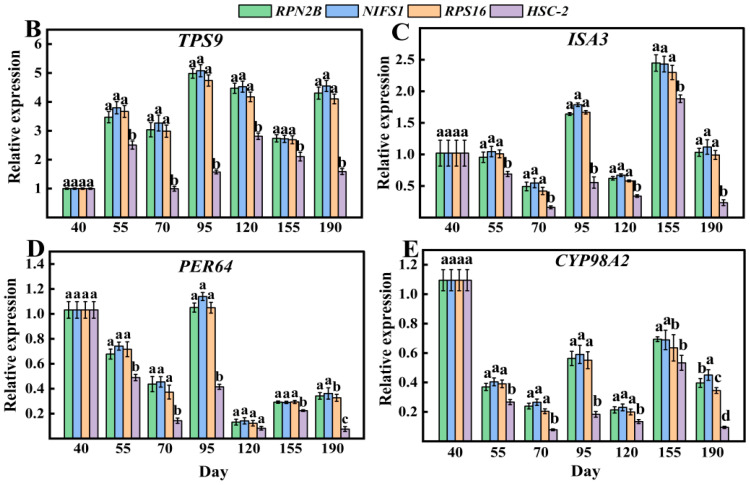
Validation of the stability of four target genes with different reference genes in Chinese olive fruits at different developmental periods. (**A**) Phenotypic characterization of Chinese olive ‘A5’ at different developmental stages; (**B**) validation in *TPS9*; (**C**) validation in *ISA3*; (**D**) validation in *PER64*; (**E**) validation in *CYP98A2*. Different letters within each column indicate significant differences between the means of the respective categories (*p* < 0.05).

The three most stable genes (*RPN2B*, *NIFS1*, *RPS16*) and one least stable reference gene (*ABCG44*) were selected for relative expression analysis of four target genes (*TPS9*, *ISA3*, *PER64*, and *CYP98A2*) across eight different varieties (Figure 8A). As illustrated in Figure 8, the relative expressions of the four target genes exhibited greater similarity and lesser variability when *RPN2B*, *NIFS1*, and *RPS16* served as standardized reference gene. Conversely, when *ABCG44* was employed as the standardized reference gene, the expression profiles of the target genes across different varieties showed increased divergence compared to the use of the other three reference genes. Additionally, the three more stable reference genes (*RPN2B*, *NIFS1*, *RPS16*) and one least stable reference gene (*HSC-2*) identified across various developmental stages were selected for relative expression analysis with the four target genes (*TPS9*, *ISA3*, *PER64*, and *CYP98A2*) at these stages (Figure 9A). As depicted in Figure 9, the expression of the target genes remained consistent when *RPN2B*, *NIFS1*, and *RPS16* were utilized as normalized reference genes. However, when *HSC-2* was applied as the normalized reference gene, the expression levels were significantly different from those observed with the other three.

## 4. Discussion

qRT-PCR is a pivotal technique for quantifying gene expression, with its accuracy significantly dependent on the stability of reference genes used for data normalization [22]. In quantitative gene expression studies, reference genes are those exhibiting stable expression across diverse experimental conditions [23]. The selection of these reference genes is crucial for ensuring data accuracy [24,25]. A stable reference gene provides a reliable baseline for quantitative gene expression analysis of gene expression, thereby enhancing the accuracy and reliability of experimental results [26]. However, when conducting validating gene expression across multiple samples, identifying a single reference gene applicable under all experimental conditions is challenging, as commonly used reference genes may be unstable [27,28,29]. Currently, no reference gene has been identified that can universally applicable across all species while maintaining consistent expression across all experimental systems. Moreover, there is no reference gene that demonstrates stable expression in every possible sample. Using inadequately validated reference genes can introduce systematic bias in experimental data [30,31]. Therefore, establishing a robust procedure for screening and validating reference genes specific to the experimental system (including species, tissue type, and treatment conditions) is essential before conducting qRT-PCR experiments [32].

Currently, commonly used reference genes in plant research include *18S rRNA*, *ACT*, *GAPDH*, *TUA*, and *TUB* [15,33,34]. Many studies employ these genes as internal controls without validating their stability in specific experimental contexts [31]. Numerous investigations have demonstrated that expression levels of these conventional reference genes vary significantly across species and conditions [35]. Therefore, identifying suitable reference genes for accurate normalization is essential. Several studies have focused on validating reference gene stability under diverse experimental conditions in plants. For example, Lv et al. [36] analyzed 14 candidate reference genes in *Zingiber officinale* across tissues, developmental stages, varieties, and abiotic stresses. Similarly, Škiljaica et al. [37] examined 10 candidates in *Arabidopsis thaliana* under varied tissues and temperatures. Zhou et al. [24] assessed seven common reference genes in *Actinidia chinensis* across varieties, tissues, developmental stages, and hormone treatments. In this study, we evaluated eight candidate reference genes in Chinese olive across varieties and developmental stages. *RPN2B* and *NIFS1* showed stable expression across all samples, confirming their suitability as reference genes. Similar validations have identified stable reference genes under stress and tissue-specific conditions in *Lentinula edodes* [38], *Malus domestica* [39]. In the research of Chinese olives, no studies have reported on the screening of reference genes, and commonly used reference genes continue to be employed. However, when validating gene expression across multiple varieties and different developmental stages of Chinese olive samples, these commonly used reference genes demonstrate unstable expression. Therefore, this study selected eight genes with relatively stable expression levels from the Chinese olive transcriptome as candidate reference genes (*RPN2B*, *PIP1.4*, *NIFS1*, *RPS16*, *At5g12110*, *HSC-2*, *ABCG44*, *LOS1*) for screening reference genes in Chinese olive fruits of various varieties and developmental stages. Based on the Cq values from qRT-PCR, multiple methods were utilized to evaluate the stability of the reference genes, including direct comparison of Cq values, geNorm, NormFinder, BestKeeper, and the online comprehensive evaluation platform RefFinder. The most stable reference genes were identified, and four target genes were used to validate the relative gene expression levels when the selected candidate reference genes were employed as standardized reference genes. The study found that, when using Chinese olives of different varieties and developmental stages as experimental materials, *RPN2B* and *NIFS1* exhibited good stability and were suitable as reference genes.

In recent years, advancements in transcriptome sequencing technology have expanded the discovery of qRT-PCR reference genes beyond conventional candidates. Screening reference genes using transcriptome data has emerged as an efficient and reliable method. For instance, Zhang et al. [40] identified eight candidate reference genes from transcriptome data and validated their suitability for *Camellia impressinervis* through rigorous analysis. In this study, we identified two novel reference genes, *RPN2B* and *NIFS1*, from Chinese olive transcriptome data. These genes provide stable reference targets that will enhance gene expression studies in this species.

## 5. Conclusions

*RPN2B* and *NIFS1* were identified as the most stable reference genes across diverse varieties and developmental stages of Chinese olive. This study establishes a robust reference gene screening system for this species, providing critical methodological support for future gene expression studies and facilitating molecular biology research in Chinese olive.

## 6. Patents

Patent application for China is in process under Application Number: 202511072675.1.

## Figures and Tables

**Table 1:** Information on Chinese olives.

Code	Name	Source	Code	Name	Source
A1	Zilaiyuan	Fuzhou, Fujian, China	A22	Gaozhou 1	Maoming, Guangdong, China
A2	Meixijianxin 2	Fuzhou, Fujian, China	A23	Wangboxianglan 1	Maoming, Guangdong, China
A3	Fulan 1	Fuzhou, Fujian, China	A24	Jiexixianglan	Jieyang, Guangdong, China
A4	Lingfeng 2	Fuzhou, Fujian, China	A25	Pingyang 2	Wenzhou, Zhejiang, China
A5	Qinglan 1	Fuzhou, Fujian, China	A26	Pingyang 4	Wenzhou, Zhejiang, China
A6	Huiyuan	Fuzhou, Fujian, China	A27	Pingyang 5	Wenzhou, Zhejiang, China
A7	Qingguo 1	Fuzhou, Fujian, China	A28	Rui’an 2	Wenzhou, Zhejiang, China
A8	Shisheng 1	Fuzhou, Fujian, China	A29	Rui’an 3	Wenzhou, Zhejiang, China
A9	Shisheng 2	Fuzhou, Fujian, China	A30	Rui’an 4	Wenzhou, Zhejiang, China
A10	Shisheng 4	Fuzhou, Fujian, China	A31	Zhuyaolan	Qinzhou, Guangxi, China
A11	Huangpichangying 26	Fuzhou, Fujian, China	A32	Niulan 1	Qinzhou, Guangxi, China
A12	Jianzhoulan	Nanping, Fujian, China	A33	Hejiang’erbaiyuan	Luzhou, Sichuan, China
A13	Zhuangbian 1	Putian, Fujian, China	B1	Grape olive	Ningde, Fujian, China
A14	Nanan 2	Quanzhou, Fujian, China	B2	Shisheng 3	Fuzhou, Fujian, China
A15	Yongdinghetou 2	Longyan, Fujian, China	B3	Nanan 1	Quanzhou, Fujian, China
A16	Sijilan 1	Ningde, Fujian, China	B4	Gaozhou 2	Maoming, Guangdong, China
A17	Suiganlan 2	Zhangzhou, Fujian, China	B5	Meixidukou 1	Fuzhou, Fujian, China
A18	Sijilan 3	Ningde, Fujian, China	B6	Zhuangbian 8	Putian, Fujian, China
A19	Lenjian	Chaozhou, Guangdong, China	B7	Pingyang 6	Wenzhou, Zhejiang, China
A20	Sihe 1	Shantou, Guangdong, China	B8	Hejiangdasuozi	Luzhou, Sichuan, China
A21	Qingpilan	Maoming, Guangdong, China			

A1–A33 are the references to screen Chinese olive varieties, and B1–B8 are the target genes to verify Chinese olive varieties.

**Table 2:** Information of 8 candidate reference genes and 4 target genes.

ID	Gene Name	Primer Sequence (5′–3)	Tm (°C)	bp
Unigene0063050	*RPN2B*	F:ATGGGTCTGCTCATGGTTGG R:GCAATGCCCAAAGCTAACCC	59.9 59.9	110
Unigene0060372	*PIP1.4*	F:TGCCAATTGGTTTCGCTGTG R:GGCATGGTCCCTGTTGAAGA	57.8 59.9	116
Unigene0041345	*NIFS1*	F:GATGGGGTTGAAGGAGGTGG R:CCACTCCCAAAGCCCTCAAA	61.9 59.9	102
Unigene0030393	*RPS16*	F:GCCTACGAGCCGATCCTTTT R:GACGGATGGCGTAGATCTGG	59.9 61.9	106
Unigene0034124	*At5g12110*	F:GTGAACCCGGGTGATTCCTT R:GCAACAATGTCACAGCTCTGG	59.9 60.8	149
Unigene0043359	*HSC-2*	F:TTGCTGGCCCTGGTGATAAG R:GTTGTCTGGGCTGTGGATGA	59.9 59.9	101
Unigene0055980	*ABCG44*	F:GATGTTCTGGGACTTGGGCA R:CCACTGTAACCCCACTGTCC	59.9 61.9	118
Unigene0053553	*LOS1*	F:CAGGCACTCGGTGAAAGGAT R:GCAGGAGACGGAAGGTGAAA	59.9 59.9	198
Unigene0011861	*TPS9*	F:TGTGGGATCTCTCAAGGCTG R:AGTCCAATATGCCCACGCTT	61.4 61.8	111
Unigene0015158	*ISA3*	F:TACTCGAGGAAGAAGCCCCT R:CTGCCACGTAGACCCAGATC	60.3 60.7	140
Unigene0017094	*PER64*	F:ATCAGAGGTTGCGATGCTTC R:TTCACCTTGTTGTGTACGGG	60.4 59.5	103
Unigene0030998	*CYP98A2*	F:GAAGTACTTGGGAGCGGTGG R:TAGTGGAGTTGGAGGGTGCA	62.0 61.7	146

**Table 3:** Amplification efficiency of the eight candidate reference genes.

ID	Gene Name	PCR Efficiency (E)	Correlation Coefficient (R^2^)
Unigene0063050	*RPN2B*	1.0267	0.9919
Unigene0060372	*PIP1.4*	0.9742	0.9994
Unigene0041345	*NIFS1*	0.9939	0.9969
Unigene0030393	*RPS16*	1.0211	0.9989
Unigene0034124	*At5g12110*	1.0361	0.9973
Unigene0043359	*HSC-2*	0.9925	0.9986
Unigene0055980	*ABCG44*	0.9697	0.9994
Unigene0053553	*LOS1*	1.0436	0.9978

**Table 4:** Coefficient of variation (CV) and standard deviation (SD) calculated by BestKeeper.

Different Varieties				Different Developmental Stages			
ID	Gene	CV	SD	ID	Gene	CV	SD
Unigene0063050	*RPN2B*	1.810	0.370	Unigene0063050	*RPN2B*	1.660	0.350
Unigene0041345	*NIFS1*	2.080	0.420	Unigene0041345	*NIFS1*	1.950	0.410
Unigene0030393	*RPS16*	2.220	0.430	Unigene0055980	*ABCG44*	2.050	0.410
Unigene0060372	*PIP1.4*	3.280	0.620	Unigene0030393	*RPS16*	2.360	0.470
Unigene0053553	*LOS1*	3.440	0.710	Unigene0053553	*LOS1*	2.630	0.550
Unigene0034124	*At5g12110*	3.470	0.730	Unigene0060372	*PIP1.4*	3.110	0.610
Unigene0043359	*HSC-2*	3.890	0.800	Unigene0034124	*At5g12110*	3.600	0.810
Unigene0055980	*ABCG44*	4.580	0.920	Unigene0043359	*HSC-2*	3.760	0.830

**Table 5:** Composite scores and rankings of candidate reference genes analyzed by RefFinder.

	Different Varieties		Different Developmental Stages	
Rank	Gene	Score	Gene	Score
1	*RPN2B*	2.000	*RPN2B*	1.000
2	*NIFS1*	2.646	*NIFS1*	2.060
3	*RPS16*	2.659	*RPS16*	2.632
4	*PIP1.4*	3.224	*LOS1*	4.427
5	*At5g12110*	3.310	*ABCG44*	5.664
6	*LOS1*	4.356	*At5g12110*	5.692
7	*HSC-2*	6.481	*PIP1.4*	6.160
8	*ABCG44*	8.000	*HSC-2*	7.113

## Data Availability

The original data presented in the study are openly available in [CRA028315, https://ngdc.cncb.ac.cn/gsa/browse/CRA028315]. The original contributions presented in this study are included in the article. Further inquiries can be directed to the corresponding author(s).
